# Cognitive disorders with epilepsy: clinical-psychopathological and neuropsychological characteristics, non-pharmacological correction

**DOI:** 10.1192/j.eurpsy.2023.991

**Published:** 2023-07-19

**Authors:** V. Korostiy, I. Blazhyna

**Affiliations:** 1 Psychiatry, narcology, medical psychology and socіal work, Kharkiv National Medical University, Kharkiv; 2 Psychiatry, Bucovina State Medical University, Chernivtsi, Ukraine

## Abstract

**Introduction:**

Cognitive dysfunction affects the development, treatment compliance, significantly worsens the quality of life and social functioning of the patients with epilepsy.

**Objectives:**

146 patients with epilepsy aged 18 to 65 participated in the study (М-40.7±2.42) were diagnosed with focal, idiopathic epilepsy and epileptic syndromes (G40.1, G40.2, G40).

**Methods:**

Clinical-anamnestic, social-demographic, clinical-psychopathological, psycho-diagnostic and statistical.

**Results:**

The study of the attention selectivity was carried out using the Munsterberg test. Only 9 examined patients (6.16%) of the total group had sufficient indices, 35 (23.97%) patients refused from the test, while the rest – 102 (69.87%) had low test results. The overall treatment group score was 7.72, which is by 13.28 lower than in the control group, where the attention selectivity index was 21 (р<0,001), which shows a considerable attention selectivity decrease in patients with epilepsy compared to the healthy persons. According to the МоСА test results, the first treatment group patients showed better cognitive functions (1.4, р<0.001), higher attention selectivity under the Munsterberg test (0.63, р<0.001), lower anxiety level under HARS (1.45, р<0.001), lower depression level under HDRS (1.7, р<0.001) and higher subjective assessment of the life quality (2.77, р<0.05). According to the МоСА test results, the second treatment group patients showed better cognitive functions (0.73, р<0.001), higher attention selectivity under the Munsterberg test (0.27, р<0,05), lower anxiety level under HARS (4.27, р<0.05), lower depression level under HDRS (2.32, р<0.05) and higher subjective assessment of the life quality (1.21, р<0.05). According to the МоСА test results, the comparison group patients demonstrated lower cognitive functions (0.22, р<0.05), higher attention selectivity under the Munsterberg test (0.15, р<0.05), lower anxiety level under HARS (2.61, р<0.001), lower depression level under HDRS (2.49, р<0.001) and higher subjective assessment of the life quality (1.0, р<0.05). The cognitive training showed its effectiveness in healthy persons of the control group: according to the МоСА test results, cognitive functions improved (0.79, р<0.001), compared to the treatment group 2 patients (0.73, р<0.001).

**Conclusions:**

According to the follow-up study data 12 months after the cognitive training and psychoeducation, follow-up study showed better values under depression and anxiety scales, and improved life quality levels in the patients of treatment groups. Patients with epilepsy show a reliable cognitive functioning improvement after a 3-month computerized cognitive training. The study results indicate a more significant cognitive functioning improvement in the patients provided the combined use of the methods of psychoeducation and cognitive training, compared to the use of a cognitive training only.

**Disclosure of Interest:**

None Declared

